# Sex difference in language cognition in the elderly group: a near-infrared spectroscopy study

**DOI:** 10.1117/1.NPh.12.1.015007

**Published:** 2025-02-12

**Authors:** Yizhu Tian, Wenyu Jiang, Mingxi Yang, Di Wu, Xiang Li, Deyu Li, Daifa Wang, Meiyun Xia

**Affiliations:** a Beihang University, School of Biological Science and Medical Engineering, School of Mechanical Engineering and Automation, Beijing Advanced Innovation Center for Biomedical Engineering, Ministry of Education, Key Laboratory of Biomechanics and Mechanobiology, Beijing, China; b Beihang University, Hangzhou International Innovation Institute, Medical Engineering and Engineering Medicine Innovation Center, Hangzhou, China; c Guangxi Jiangbin Hospital, Department of Neurological Rehabilitation, Nanning, China; d Chinese Academy of Sciences, Shenzhen Institutes of Advanced Technology, Shenzhen, China; e Beihang University, State Key Laboratory of Virtual Reality Technology and System, Beijing, China

**Keywords:** sex difference, language cognition, verbal fluency, functional near-infrared imaging

## Abstract

**Significance:**

There are sex differences in the incidence and prevalence of cognitive disorders, such as Alzheimer’s disease. Whether this difference is already present in the preclinical stage of the disease is unclear.

**Aim:**

We aim to explore whether there are sex differences in brain functional activities of specific cognitive tasks in the elderly and identify sex-related biomarkers of specific cognitive functions, which may provide important references for the mechanism disclosure and clinical early screening and diagnosis of cognitive disorders.

**Approach:**

We measured global cerebral hemoglobin concentrations and connectivity in elderly male (n=45) and female (n=44) groups during the letter and category verbal fluency tasks. The sex differences in activation and connectivity and their relationship with task performance were explored.

**Results:**

We found that there is a significant sex difference in connectivity, especially connectivity between the left inferior parietal and right prefrontal and left and right occipital in letter tasks, including the connectivity in parietal, left inferior parietal, and left occipital in category tasks. These connectivities were also significantly negatively correlated with the task performance of male groups.

**Conclusions:**

Our results indicated the connectivity between the left inferior parietal and right prefrontal, left and right occipital in letter tasks; the internal connectivity in the parietal; and the connectivity between parietal and the left inferior parietal and right occipital in category tasks may be crucial for verbal assessment of aging males. It is expected that the results will assist in cognitive assessment in the elderly group.

## 1 Introduction

Neurobiological studies of population-based samples have identified sex differences in brain structure and function, many of which may be exacerbated in abnormal brain networks, such as neurological disorders.^1^^,^^2^ Sex differences have been reported in the incidence and prevalence of cognitive disorders (e.g., Alzheimer’s disease).^3^ Still, sex differences in the elderly group remain understudied, particularly in the preclinical stages of the disease, which can provide a baseline for the identification of abnormal brain networks in the disease state. Exploring whether there are sex differences in brain functional activities of specific cognitive tasks in the elderly and identifying sex-related biomarkers of specific cognitive functions can provide important guidance on risk factors, pathogenesis, and personalized intervention treatment of cognitive disorders.

Language impairment is often among the early symptoms of cognitive decline in the elderly.^4^^–^^6^ The language-related tasks, such as verbal fluency, are also commonly used for age-related research and neurodegenerative disease assessment.^7^^–^^9^ In addition, language processes are typically referred to sex differences. A quite consistent female superiority has been observed in verbal fluency and verbal memory abilities reported by previous studies.^10^^–^^13^ Previous studies have reported that healthy adults show sex differences in language-related activation,^14^^–^^17^ brain structural network efficiency, and functional network.^18^^–^^20^ Females are considered to have higher regional efficiency in the left superior parietal and superior temporal and greater overall cortical connectivity and interhemispheric connectivity in bilateral but decreased local connectivity, whereas males have a greater tendency to connect within regions during language processing.^21^^,^^22^ Sex-related studies have shown that males have a significant left lateralization activation in the inferior frontal gyrus, whereas females tend to have bilateral activation in language processing.^23^^,^^24^ This lateralized difference is also reflected in functional connectivity patterns, with females having more interhemispheric connectivity in bilateral areas.^21^^,^^22^ Several studies discussed the influence of both age and sex, although most considered them as two independent variables. The sex differences in volume loss observed in frontal, temporal, and parietal regions are discussed in neuroanatomical studies.^25^ Older males showed a tendency toward relatively increased activation in the frontal, temporal, and left supramarginal gyrus, whereas females exhibited an increase in bilateral inferior frontal junction activation.^26^^,^^27^ There are also differences in the network organization. Previous studies revealed a reduction in overall cortical connectivity and a shift of regional efficiency from the lower (i.e., occipital) to the upper (i.e., frontal). Females showed greater overall connectivity, both locally and globally.^28^^–^^30^ Whether there exist sex-related differences in language cognitive functions in the elderly also needs further investigation.

The verbal fluency test (VFT) serves as a quick and effective screening tool for assessing language ability.^31^^–^^33^ VFT is often included in neuropsychological, clinical, and non-clinical assessments.^34^ The tasks require subjects to generate as many words as possible under specific conditions (orally or written) and within time constraints while avoiding repetition and proper nouns.^35^ The generation criteria are either semantic, e.g., naming animals and fruits, known as category fluency, or phonemic, e.g., naming words that begin with a specific letter, also called letter fluency. Although these two VFTs share several core processes, such as attention, and inhibition of repeated words, they differ concerning the kind of search processes. Phonemic fluency may involve a serial search based on the syllabification of given letters. Category fluency is driven by association chains and extends to related subcategories, involving actively shifting between generation categories or active subcategories.^36^

Functional near-infrared spectroscopy (fNIRS) is an emerging optical neuroimaging technique that monitors brain activity by measuring oxygenated hemoglobin (HbO) and deoxygenated hemoglobin (HbR) concentrations. Owing to its unique technical advantages, such as robustness to motion, a wide range of applicable populations, and environment friendliness, fNIRS is widely used in the study of brain function in VFT.^37^^–^^39^

There are sex differences in the incidence and prevalence of cognitive disorders. Whether this difference is already present in the preclinical stage of the disease is unclear. Our study aimed to explore whether there are sex differences in task-related brain functional networks and identify and determine the differences in functional networks between male and female groups in specific cognitive functions. It can provide important information for cognitive assessment and personalized treatment of cognitive impairment in the elderly group. To preliminarily understand the sex differences in cognitive function in the elderly group and then provide assistance for early cognitive assessment of sex-specific groups, this study used fNIRS technology to explore the sex differences in brain activity during VFT tasks in the elderly group.

## 2 Method

### 2.1 Subjects

A total of 100 subjects over the age of 60 were recruited from the Guiya community in Nanning of Guangxi Zhuang Autonomous Region. Of these, 89 elderly subjects were included in the subsequent analyses (Table 1). Subjects were excluded because of their unavailable fNIRS data (poor signals/artifacts in the preprocessing of fNIRS, n=8) or the failure to complete fNIRS recording (n=3). All the subjects were right-handed and native Chinese speakers. None of the subjects had pre-existing neurological or psychiatric disorders.

**Table 1:** Demographic information of subjects.

	Age (year)	Education (year)	MoCA
Overall	67.685±4.601	11.292±2.638	26.742±2.309
Male (n=45)	68.089±4.236	11.533±2.710	26.867±2.063
Female (n=44)	67.273±4.962	11.046±2.570	26.614±2.554
p (male versus female)	0.406	0.386	0.608

MoCA, Montreal cognitive assessment.

### 2.2 Experimental Paradigms

**Fig. 1:**
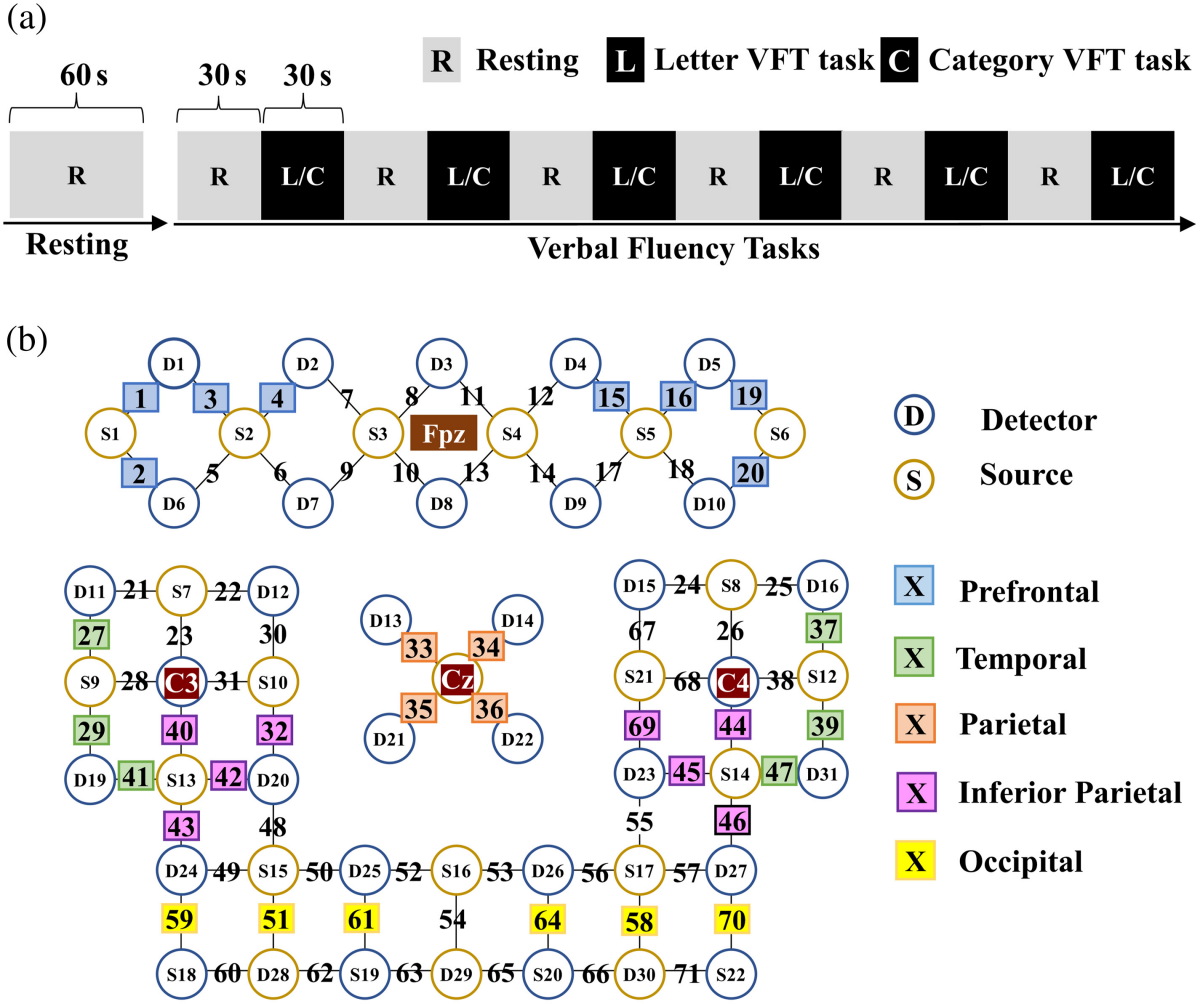
Experiment settings: (a) the paradigms: resting and task period and (b) arrangement of the fNIRS probes and channels of ROI.

The experiment paradigm included the resting state and the VFT [Fig. 1(a)]. The resting state lasted for 60 s. During the resting state, the subjects were asked to sit comfortably in a chair in front of a computer screen, keep quiet, and not move. The purpose of resting paradigm design is to help subjects adjust their states and be ready for the execution of later tasks. The VFT consisted of six consecutive blocks, three letter VFT blocks, and three category VFT blocks, each block had 30 s of rest and 30 s of the task, which was ∼6  min. During the letter VFT task, participants were asked to pronounce as many Chinese nouns as possible beginning with specific Chinese characters (“大,” “上,” “光”) without proper names and repetition. During the category VFT task, they had to generate words that belonged to a particular semantic category (“animals,” “fruit,” “ball sports”) without proper names and repetition.^40^ The six blocks were presented randomly.

### 2.3 Functional Near-Infrared Spectroscopy

Hemodynamics was measured with two wavelengths of near-infrared light (i.e., 760 and 850 nm) by fNIRS machines (Nirscan, Huichuang, China). According to the international 10 to 20 system, the 22 source and 31 detector probes were fixed and placed on the brain of the subject. The arrangement is shown in Fig. 1(b). The midpoint between S3 and S4 was Fpz. S11, D17 and D18 were Cz, C3 and C4, respectively. The distance between pairs of source and detector probes was 3 cm, and the sampling rate was 19 Hz.

The regions of interest (ROIs) as shown in Fig. 1(b),^41^^,^^42^ the left prefrontal lobe (LF, channels 1 to 4), right prefrontal lobe (RF, channels 15, 16, 19, 20), parietal lobe (P, channels 33 to 36), left temporal lobe (LT, channels 27, 29, 41), right temporal lobe (RT, channels 37, 39, 47), left inferior parietal lobule (LIP, channels 32, 40, 42, 43), right inferior parietal lobule (RIP, channels 44, 45, 46, 69), left occipital lobe (LO, channels 51, 59, 61), and right occipital lobe (RO, channels 58, 64, 70).

### 2.4 Data Preprocessing

The resting stage in experimental paradigms was mainly used for state adjustment of subjects. Our data processing and analysis only included the data during VFT tasks.

The preprocessing of fNIRS data was completed by Homer2. Motion artifacts were amended by spline interpolation methods (AMPThresh = 3, TMotion = 0.5 s, TMASK = 1 s, and STDEVthresh = 20).^43^ Then, physiological noise was removed by a bandpass filter between 0.1 and 0.01 Hz.^44^ The modified Beer–Lambert law was used to convert optical signals to concentration signals. The differential path-length factor was 6. The baseline drift was corrected by a first-order linear fitting.^45^

For the task session, we calculated brain activation, internal and interregional ROI connectivity of channels for letter, and category VFT tasks by averaging the data in its corresponding three blocks. ROI activation was the average of all channel activations in the region. The functional connectivity of any two channels was calculated by Pearson correlation and then normalized by Fisher’s r-to-z transformation. The internal ROI connectivity was averaged by all connectivity of channels within the ROI, and the interregional ROI connectivity was averaged by all functional connectivity of channels between two ROIs.

### 2.5 Statistical Analysis

For behavior performance, the number of correct, intrusion, and perseveration words for the letter VFT and category VFT was recorded for the male and female groups. The two-sample independent t-test was used to compare the differences between the two groups. The one-sample or two-sample t-test was conducted for normally distributed data and the Wilcoxon signed-rank test or the Mann–Whiney U-test for non-normal distribution data.

To investigate the relationship between subjects’ performance (the number of correct words) and their brain activity (activation and connectivity),^46^ we performed correlation analyses between the brain activity (ROI activation, internal ROI connectivity, and interregional ROI connectivity) and the number of words obtained in VFT task. The Pearson or Spearman correlation coefficient was used for normal distribution and non-normal distribution, separately.

All t-tests were corrected using a false discovery rate (FDR). When p<0.05, the difference was considered statistically significant.

## 3 Results

A total of 89 subjects were included in the data analysis and discussion. Before data preprocessing, three subjects were excluded due to their incomplete performance of VFT tasks. During data preprocessing, five subjects had poor fNIRS signals on more than half of the channels; three subjects had multiple, prolonged, large body movements during the task; and the results of motion artifact removal were not satisfactory. A total of eight subjects were excluded. After data preprocessing, all the data of 89 subjects were statistically analyzed in our study, including three blocks for the letter tasks and three blocks for the category tasks.

### 3.1 Task Performance

**Fig. 2:**
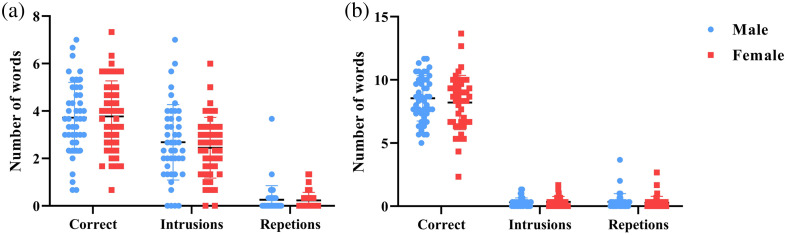
Task performance: (a) letter VFT task and (b) category VFT task.

The behavioral results are shown in Fig. 2. There was no significant difference between the male and female groups.

### 3.2 Functional Near-Infrared Spectroscopy

**Fig. 3:**
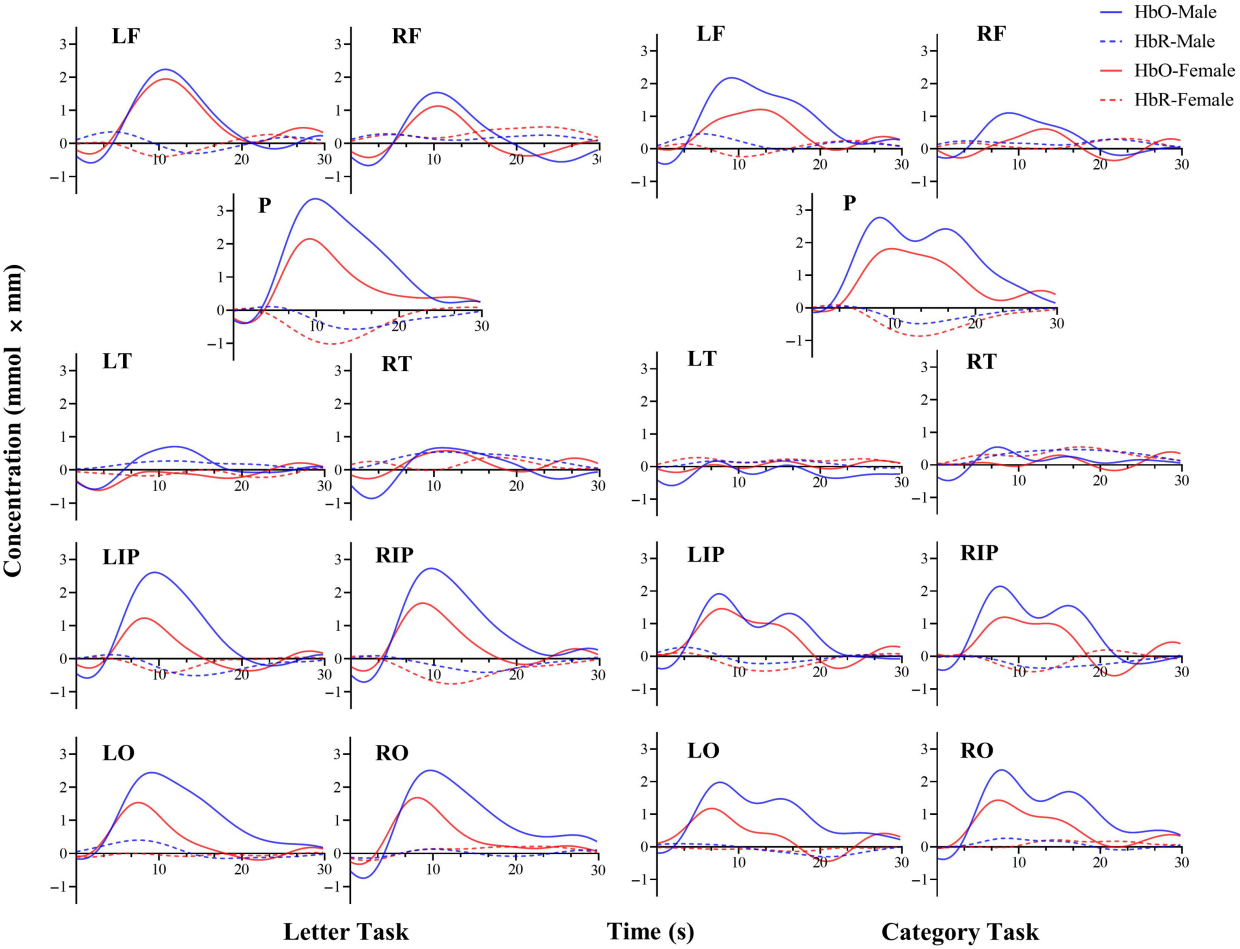
Activation curves in the VFT tasks.

Task activation was shown in Fig. 3. For the category VFT task, the male group showed significantly higher HbO concentrations in the parietal (p=0.044) and the left occipital (p=0.033). After FDR correction, the significance disappeared. In addition, there was no significant difference between the two groups in the category of VFT tasks based on HbR activation. There was also no significant difference between groups in the activation of letter VFT tasks, whether based on HbO or HbR.

**Fig. 4:**
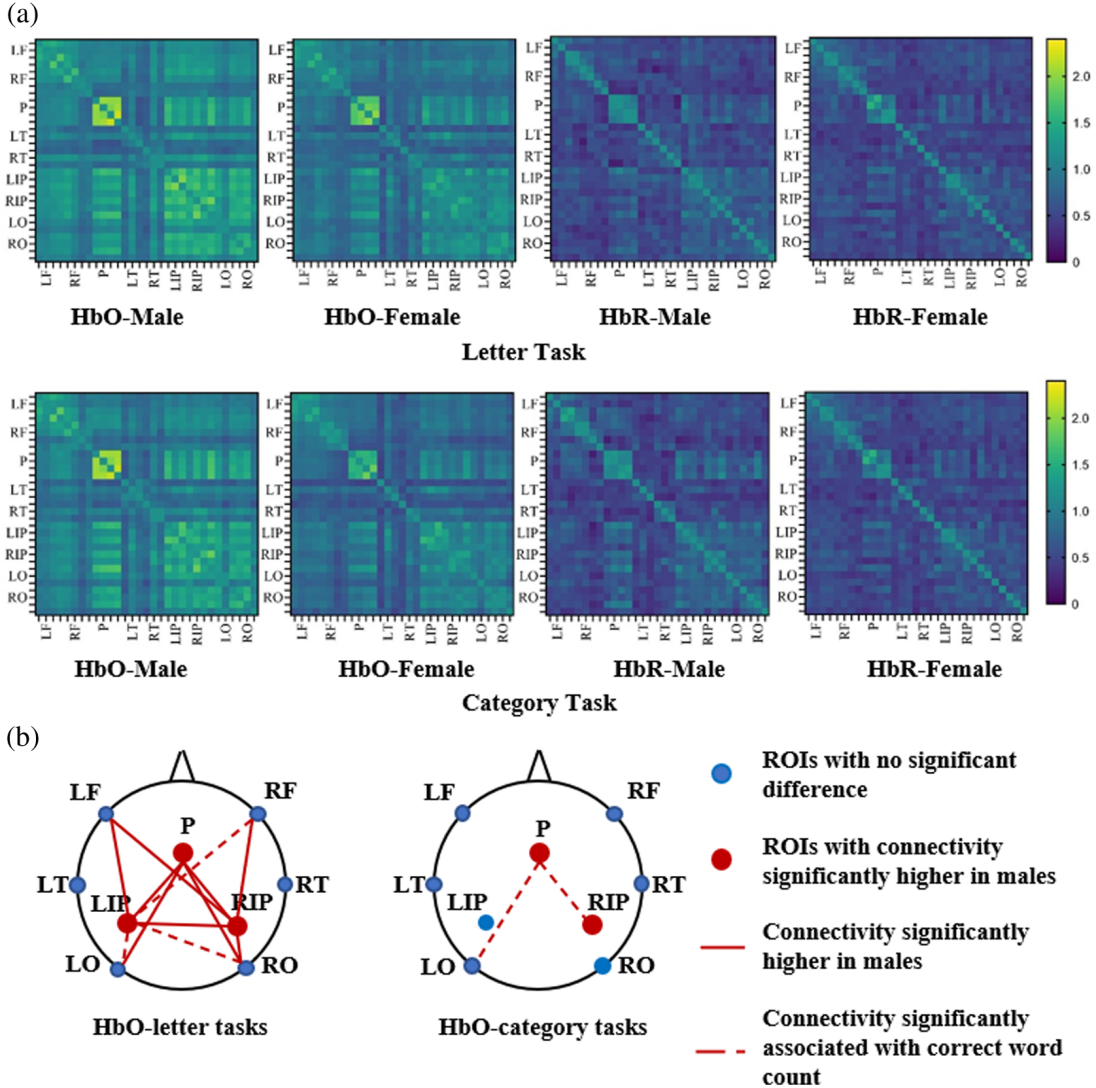
Connectivity and correlation results: (a) male and female connectivity in the VFT tasks and (b) male connectivity with significant difference and correlation in letter tasks.

As shown in Fig. 4(a), the male group had significantly higher whole-brain functional connectivity in these two VFT tasks than the female group (letter task: p=0.004, category task: p=0.006). Figure 4(b) shows the differences in HbO connectivity intra- and inter-ROIs between the two groups of subjects. In the letter VFT task, the connectivity in the parietal (p<0.001) and left (p<0.001) and right (p<0.001) inferior parietal was significantly higher in the male group than the female group [left in Fig. 4(b)]. The functional connectivity associated with the parietal lobe, the left inferior parietal lobe, and the right inferior parietal lobe was significantly greater in the male group than the female group (see Table 2). For category VFT tasks, intra-parietal (p<0.001) and right inferior parietal (p=0.011) connectivities were significantly higher in males than females [right in Fig. 4(b)], and P-LO and P-RIP connectivities also showed significant sex differences. As for functional connectivity analysis based on HbR concentration, no significant differences between groups were found. The above results were corrected by FDR. These results suggest that for VFT tasks, especially letter VFT tasks, sex differences in functional connectivity between men and females are mainly concentrated in the posterior brain.

**Table 2:** Connectivity with significant sex differences.

Letter VFT task		Category VFT task	
HbO connectivity	p (male versus female)	HbO connectivity	p (male versus female)
P-P	<0.001	P-P	<0.001
LIP-LIP	<0.001	—	—
RIP-RIP	<0.001	RIP-RIP	0.011
LF-LIP	<0.001	—	—
LF-RIP	0.003	—	—
RF-LIP	0.010	—	—
RF-RIP	0.011	—	—
P-LIP	0.006	—	—
P-RIP	0.002	P-RIP	<0.001
P-LO	0.008	P-LO	0.001
P-RO	0.002	—	—
LIP-RIP	<0.001	—	—
LIP-LO	<0.001	—	—
LIP-RO	0.002	—	—
RIP-RO	0.007	—	—

### 3.3 Correlation Analysis

Based on the results of the sex differences in brain activity described above, the study found that only male subjects had a significant negative correlation between functional connectivity and behavioral performance [see Table 3 and Fig. 4(b)]. In the letter VFT task, the functional connectivity associated with LIP, such as LIP-RF, LIP-LO, and LIP-RO, was significantly negatively correlated with the correct number of words. In the category VFT task, functional connectivity related to the parietal lobe, such as intra-parietal connectivity (P-P), and interparietal connectivity (P-RIP and P-LO), was significantly negatively correlated with the correct number of words. These results suggest the potential importance of LIP and P in letter and category VFT tasks, respectively.

**Table 3:** Connectivity with significant correlations in task performance.

Letter VFT task—male group			Category VFT task—male group		
HbO connectivity	Number of correct		HbO connectivity	Number of correct	
	r	p		R	P
RF-LIP	−0.296	0.049	P-P	−0.341	0.022
LIP-LO	−0.369	0.013	P-RIP	−0.342	0.021
LIP-RO	−0.314	0.036	P-LO	−0.392	0.008

## 4 Discussion

To preliminarily investigate the sex differences in language cognitive function in the elderly group and provide references for clinical evaluation of neurocognitive disorders, this study explored whether there are sex differences in brain functional activities of VFT tasks in the elderly group and suggested possible sex-specific features. Brain activity during letter and category VFT tasks was measured by fNIRS in 45 males and 44 females, and task-related HbO/HbR hemodynamic responses were analyzed. The study found no significant differences between males and females in terms of behavior and HbO-based brain region activation but showed significant intergroup differences in functional connectivity. Among them, in the letter task, the LIP-RF, LIP-LO, and LIP-RO connectivities of the male group increased significantly and were significantly correlated with the number of correct words. In the category task, the male group had significantly higher P-P, P-RIP, and P-LO connectivities and a significant correlation with the correct word count. Generally, the HbO concentrations showed more significant differences between groups, which is in line with the previous literature suggesting the sensitivity to task-associated change of HbO.^47^ Therefore, in the following discussion, we mainly focused on the HbO results.

There were no significant sex differences in task performance and, correspondingly, no significant intergroup differences in activation. However, there were significant sex differences in connectivity, which were significantly correlated with task performance in the male group.

We found that the connectivity with parietal and inferior parietal showed significant sex differences in tasks. Previous functional magnetic resonance imaging (fMRI) studies showed that there are sex differences in the default mode network (DMN) connectivity of parietal nodes (including posterior cingulate cortex and angular gyrus) and precuneus at resting state.^48^^,^^49^ Compared with the resting state, the sex difference network of the task state overlaps, but there are differences. Except for brain regions with significant sex differences in resting states (parietal and inferior parietal), there was a broader network of sex differences in letter tasks, including connectivity between the inferior parietal and bilateral prefrontal, parietal, or occipital lobes. The parietal and inferior parietal are important language processing areas involved in the association and integration of language information.^50^^,^^51^ This regional sex difference may be related to recognition strategies. In letter tasks, switching, the ability to shift between lexical clusters, has a high correlation with the number of words produced and,^52^ on the other hand, usually means more internet connectivity. In addition, females generally have more interhemispheric connectivity, whereas males tend to be more localized in local connectivity.^22^ Thus, in letter tasks, multiple interhemispheric connectivity showed sex differences. Compared with category tasks, letter tasks showed additional left inferior parietal–related sex difference connectivity, which may be related to category-switching selection or phonemic language processes.^53^^,^^54^

In addition, the LIP-RF, LIP-LO, and LIP-RO connectivities in letter tasks and the intra-parietal, P-RIP, and P-LO connectivities in category tasks were significantly negatively correlated with the task performance of male groups. These differences in connectivity may represent a compensatory mechanism in older males to maintain performance in different language tasks. The parietal, inferior parietal, and occipital lobes are thought to be part of the extended language system.^51^ In letter tasks, the connectivity with significant correlation was concentrated in the left inferior parietal and involved the right frontal and occipital lobes. Previous studies have found involvement of additional brain regions in the Broca (prefrontal) and Wernicke (inferior parietal) areas in the aging group in phonemic fluency tasks and that functional connectivity of the Wernicke area is correlated with higher performance in phonemic fluency.^55^ Although the Wernicke area has traditionally been associated with language comprehension, rather than language production, a possible explanation is that the Wernicke area may also involve knowledge about the sequence of consonant and vowel speech sounds (phonemes) and phonologic retrieval.^54^ Changes in the relevant connectivity in this region may indicate the recruitment of additional language networks, specifically the connecting brain regions, the right frontal and occipital, which are also reported to be the important component in phonemic word generation,^52^ and extra activated in older adults during language tasks.^56^^,^^57^ In category tasks, parietal-related connectivity showed a significant negative correlation. Category tasks are more dependent on semantic association processes, whereas the parietal is thought to play an important role in semantic memory and relevant information selection, and activity was negatively varied with task difficulty in the semantic tasks.^58^ The connectivity with the occipital showed a significant correlation in both VFT tasks. This may indicate visual-related compensatory processes (e.g., visual imagery) in the male group,^59^ whereas males are generally reported to have a visual-spatial advantage.^60^ Therefore, we suggested that interregional connectivity in LIP-RF, LIP-LO, and LIP-RO for letter tasks and internal connectivity in the parietal, interregional connectivity in P-RIP, and P-LO for category tasks may deserve more attention in clinical evaluation.

Several limitations associated with the present study should be noted. Throughout the life cycle, the structure and function of the brain change at different stages and may show different patterns of sex differences. This paper is an exploratory study that presents a cross-sectional analysis of cognitive ability in the elderly population. In future studies, we will further analyze the long-term cognitive development process of the elderly group and compare patterns of male and female brain activity at different life stages. This can help us better understand the laws of sex-related cognitive development and disease occurrence and development and provide objective and quantitative brain imaging indicators for early diagnosis and treatment of specific diseases. In addition, this study was an exploratory study based on small sample size, and a multi-center controlled study based on a large sample size should be conducted in the future to refine the effects of age and education levels on the cognitive function of different sex groups. Furthermore, in this study, we mainly focus on sex differences in language tasks in older age groups and explore specific biomarkers to provide references for early geriatric cognitive screening in clinical. In the future, based on this study, we will compare the sex difference network between resting and task state and carry out exploratory research with a larger sample size for more cognitive functions. We will also establish a cognitive assessment and analysis model for the whole life cycle to reveal the influencing factors of cognitive decline from multiple perspectives.

## 5 Conclusion

In conclusion, the present study demonstrated that elderly subjects showed sex differences in brain connectivity in letter VFT tasks. The connectivity with the inferior parietal and the parietal was significantly correlated with task performance in the male group for letter and category tasks, respectively. By further including the between-groups analysis during the resting state and verifying with a larger sample size, it is expected that the results will provide an important reference for exploring the influence of sex on mechanism interpretation and clinical evaluation.

## Data Availability

The data that support the findings of this paper are not publicly available due to privacy or ethical restrictions. They can be requested from the corresponding author.
